# Structural organization of erythrocyte membrane microdomains and their relation with malaria susceptibility

**DOI:** 10.1038/s42003-021-02900-w

**Published:** 2021-12-08

**Authors:** Anna Olivieri, Rebecca S. Lee, Federica Fratini, Cyrianne Keutcha, Mudit Chaand, Valentina Mangano, Francesco Celani, Stefania Mochi, Cecilia Birago, Silvio Paone, Felicia Grasso, Valentina Tirelli, Mario Falchi, Estela Shabani, Stefania Bertoncini, Bienvenu Sodiomon Sirima, Elisabetta Pizzi, David Modiano, Manoj T. Duraisingh, Marta Ponzi

**Affiliations:** 1 Dipartimento di Malattie Infettive, Istituto Superiore di Sanità, Rome, Italy; 2 Department of Immunology and Infectious Diseases, Harvard T. H. Chan School of Public Health, Boston, MA USA; 3 Servizio Grandi Strumentazioni e Core Facilities, Istituto Superiore di Sanità, Rome, Italy; 4 Dipartimento di Sanità Pubblica e Malattie Infettive, Sapienza Università di Roma, Rome, Italy; 5 Dept. of Traslational Research, University of Pisa, Pisa, Italy; 6 National HIV/AIDS Research Center (CNAIDS), Istituto Superiore di Sanità, Rome, Italy; 7 Department of Biology, University of Pisa, Pisa, Italy; 8 Centre National de Recherche et de Formation sur le Paludisme (CNRFP), Ouagadougou, Burkina Faso

**Keywords:** Parasite host response, Malaria

## Abstract

Cholesterol-rich microdomains are membrane compartments characterized by specific lipid and protein composition. These dynamic assemblies are involved in several biological processes, including infection by intracellular pathogens. This work provides a comprehensive analysis of the composition of human erythrocyte membrane microdomains. Based on their floating properties, we also categorized the microdomain-associated proteins into clusters. Interestingly, erythrocyte microdomains include the vast majority of the proteins known to be involved in invasion by the malaria parasite *Plasmodium falciparum*. We show here that the Ecto-ADP-ribosyltransferase 4 (ART4) and Aquaporin 1 (AQP1), found within one specific cluster, containing the essential host determinant CD55, are recruited to the site of parasite entry and then internalized to the newly formed parasitophorous vacuole membrane. By generating null erythroid cell lines, we showed that one of these proteins, ART4, plays a role in *P. falciparum* invasion. We also found that genetic variants in both *ART4* and *AQP1* are associated with susceptibility to the disease in a malaria-endemic population.

## Abstract (web summary)

Olivieri et al. exploit floating properties of microdomain-associated proteins to investigate host proteins important for Plasmodium falciparum erythrocyte invasion. Using proteomic and bioinformatic approaches, they analyze clusters of protein abundance profiles from detergent resistant membranes (DRMs) of erythrocytes and identify a host protein, ART4, important for P. falciparum invasion into RBCs.

## Introduction

Plasma membranes are compartmentalized into microdomains exhibiting distinctive lipid and protein composition, size, and dynamics, also referred to as lipid rafts. These membrane assemblies, virtually present in all cell types, are enriched in cholesterol and sphingolipids and function as real-sorting domains, where protein composition may change in response to stimulation^1^. Membrane microdomains were shown to be involved in various biological processes, such as signaling via receptors, intracellular trafficking, cellular differentiation, and infection by intracellular pathogens, including bacteria, viruses, and parasites^2^. In particular, it was suggested that erythrocyte microdomains could play a role in susceptibility to malaria infection^3^.

Malaria is one of the most deadly diseases worldwide, with 228 million cases and 405 thousand lethal outcomes in 2018^4^. The burden is heaviest in Africa, where more than 90% of all malaria deaths occur, mostly in children under 5 years of age.

The vast majority of the lethal events due to malaria are caused by the parasite *Plasmodium falciparum*. This pathogen develops and replicates inside hepatocytes during the silent phase of infection and inside erythrocytes during the clinical phase. The invasive forms, the merozoites, infect human erythrocytes via a complex multi-step process. Once inside the erythrocytes, the trophic forms, the trophozoites, grow and multiply within a parasitophorous vacuole (PV). Upon maturation, daughter merozoites emerge from the host cell, free to invade new erythrocytes.

An essential role of erythrocyte membrane microdomains in susceptibility to invasion by the malaria parasite *P. falciparum* was suggested by the reports that modification^5^ or disruption^3^ of these subcellular compartments prevent invasion by merozoites. In infected erythrocytes, host membrane microdomain proteins were shown to be recruited to the PV membrane (PVM), suggesting that internalization occurs during invasion^6^. Despite the proved role of host cholesterol-rich microdomains in malaria pathogenesis, only a few proteins associated with these membrane compartments have been characterized so far^6^.

By using a novel approach recently developed by the authors^7^, we performed a comprehensive quantitative proteomic analysis of erythrocyte membrane microdomains and grouped the most represented proteins in 9 clusters on the basis of their buoyancy profiles. This functional compartment includes the vast majority of erythrocyte proteins known to be involved in *P. falciparum* invasion.

We focused in particular on cluster 3, containing the blood group CD55 and two proteins previously described as high-ranking candidates for being involved in invasion, Ecto-ADP-ribosyltransferase 4 (ART4) and Aquaporin 1 (AQP1)^8^. We showed that ART4 and AQP1 coalesce in proximity to the parasite entry site upon invasion, suggesting an infection-dependent remodeling of erythrocyte membrane microdomains.

By generating null erythroid cells, we showed that ART4 plays an important role in erythrocyte invasion by *P. falciparum*, while AQP1 is dispensable. Moreover, genetic variations at both the *ART4* and *AQP1* loci are significantly associated with severe malaria and parasite density in children from a Sub-Saharan African malaria-endemic country. Together, we found that multiple proteins associated to an erythrocyte microdomain type, are implicated in aspects of malaria pathophysiology.

## Results

### Proteomic analysis of erythrocyte detergent-resistant membranes

Aim of this work is to provide a wide and comprehensive analysis of membrane microdomains of human red blood cells (RBCs). Biochemical characterization of cholesterol-rich membrane microdomains relies on their resistance to solubilization by certain non-ionic detergents at low temperature, which allows to isolate them as detergent-resistant membranes (DRMs) by sucrose gradient centrifugation.

To reduce variability due to inter-individual differences, fresh RBCs were obtained from the pooled blood of 7 healthy donors and DRMs were independently isolated from six stored samples. Twelve fractions were collected from each of the six gradients: low-density fractions (2–8), containing DRMs, and heavy-density fractions (9–12), containing detergent soluble membranes^7^.

Effectiveness and reproducibility of DRM separation were assessed by probing the low-density fractions 2–8 of each gradient with an antibody against the raft-associated protein Flotillin-1 (Supplementary Fig. S1), mainly floating to fraction 4 in our experimental conditions^7^. Proteins in low-density fractions were then separately analyzed by mass spectrometry. A total of 201 proteins were identified, 93.6% of which were also detected in two recent erythrocyte proteomic analyses (Supplementary data 1 and 2)^9,10^.

To define the floating features of RBC DRMs and their relation with malaria disease, we selected the 147 proteins detected in at least three out of six preparations, likely corresponding to abundant DRM-associated proteins, for further analysis. Abundance values assigned to each protein identified in the low-density fractions were used to generate protein abundance profiles (PAPs)^7^. To evaluate the reproducibility of PAPs between replicates, we calculated Pearson’s correlation values (*R*) for each PAP pairs. The number of correlation coefficients ranged between 3 and 15, depending on the number of replicates each protein was identified in. 71% of DRM-associated proteins displayed conserved PAPs (*R* ≥ 0.6 in more than 50% profile pairs; Supplementary Fig. S2), suggesting that the adopted procedure is robust and reliable. The presence of a protein subset displaying less conserved PAPs (29%) may be explained by the existence of DRM-associated proteins residing in membrane contexts partially susceptible to detergent extraction.

#### RBC DRMs are heterogeneous in buoyancy properties and functional annotation of protein components

**Fig. 1:**
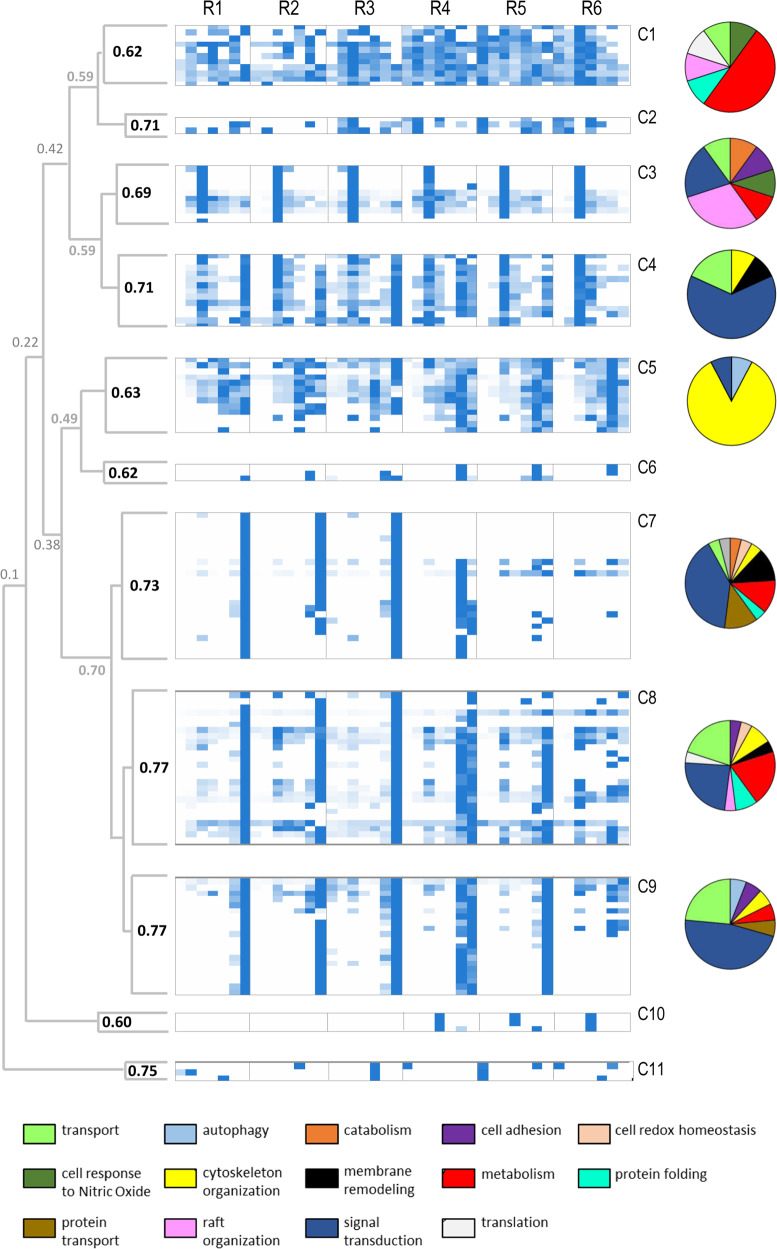
Hierarchical clustering of PAPs. Clustering of PAPs from six replicates (R1–R6) of human erythrocyte DRMs is shown as a heatmap. Dendrogram architecture and Pearson’s correlation coefficients corresponding to principal nodes are also reported. Pearson’s correlation values for clusters C1–C9, with coefficients *R* ≥ 0.6 (*P* ≤ 0.005), are shown in black. Protein functional assignment in clusters with more than three members is shown as pie charts. Shades of blue relate to the relative abundance values of the identified proteins.

To describe the overall organization of RBC DRMs, we merged protein-related PAPs from each replicate in single meta-profiles and submitted them to hierarchical average linkage clustering. Protein groups, based on profile similarity, were defined according to dendrogram architecture using as a threshold a correlation coefficient *R* ≥ 0.6 (*P* ≤ 0.005). As shown in Fig. 1 and Supplementary Data 3, DRM-associated proteins were categorized into nine clusters.

Interestingly, we observed an uneven distribution of profile conservation between clusters (Supplementary Fig. S2), as well as a segregation of functional pathways, as assigned by Gene Ontology (GO) (Fig. 1 and Supplementary Data 3). For instance, Cluster 1, with profiles variable between replicates, contains an abundance of proteins involved in a broad range of metabolic pathways, most likely transiently recruited to membrane microdomains or associated with a less ordered lipid environment. Cluster 1 also includes Annexin A2, involved in both organizing lipid rafts and linking them to cytoskeletal components^11^. Annexins are not structural membrane components as their binding to phospholipids is regulated by calcium signaling^11^. Cluster 2 is composed of proteins with highly conserved profiles. Consistently, it includes the structural components of erythrocyte lipid rafts Flotillin-1 and Flotillin-2, and the myeloid-associated differentiation marker (MYADM) also described as a raft organizer^12^. Cluster 3 contains mainly signal transduction proteins, including potential actors of the RBC apoptotic process, referred to as eryptosis^13^. These include Calpain 5, AQP1, Scramblase 4, and the Guanine Nucleotide-binding protein G(i) subunit alpha-2. RBC DRMs have been shown to be associated with the underlying membrane skeleton^14^. Consistently, we detected skeletal proteins, including components of the junctional complex, mainly confined to cluster 4. This cluster also includes the junctional complex component Membrane Palmitoylated Protein 1 (MPP1/p55) that also functions as a raft organizer^15^. The raft-scaffolding protein Stomatin^16^ and its interactors^17^ are all grouped in the closely-related clusters 7 and 8 (*R* = 0.75).

First conclusions about the DRM analysis at a whole-cell level are: (i) protein floating properties could be related not only to the spatial but also to the functional organization of erythrocyte membrane; (ii) protein groups containing Flotillins and Stomatin, major raft organizers of erythrocyte, exhibit different flotation properties and protein composition, likely identifying distinct microdomains, as previously suggested^18^.

#### Erythrocyte membrane microdomains in relation to malaria parasite infection

Interestingly, we found that erythrocyte DRM proteome includes the majority of proteins known to play a role in *P. falciparum* invasion: Semaphorin-7A^19^, protein G subunit alpha-s^20^, the blood group CD55^8^, Glycophorin A, and C^21^, Basigin receptor (BSG)^22^, the ATP-binding cassette sub-family B member 6 (ABCB6)^23^, and the Ras-related C3 botulinum toxin substrate 1 (Rac1)^24^.

It was suggested that parasites exploit the host signal transduction machinery to invade erythrocytes^20^, a mechanism common to several intracellular pathogens^25^. Noteworthy, cluster 3, comprising 13 proteins, includes several members of the classical signal transduction cascade. It also includes two proteins previously indicated by Egan and co-authors as high-ranking candidates as markers of parasite invasion, ART4 and AQP1^8^.

Cluster 3 proteins were identified in fraction 4, where raft organizers reside, and in fraction 8, at the boundary with DSMs, suggestive of a dynamic association to lipid rafts. These buoyancy features were confirmed on a new RBC-DRM preparation from an independent pool of 7 donors, by probing gradient fractions 2–8 with antibodies against CD55 and AQP1 (Supplementary Fig. S3). Flotillin-1, used as a control, peaks at fraction 4 as expected.

#### Subcellular localization of proteins belonging to cluster 3

**Fig. 2:**
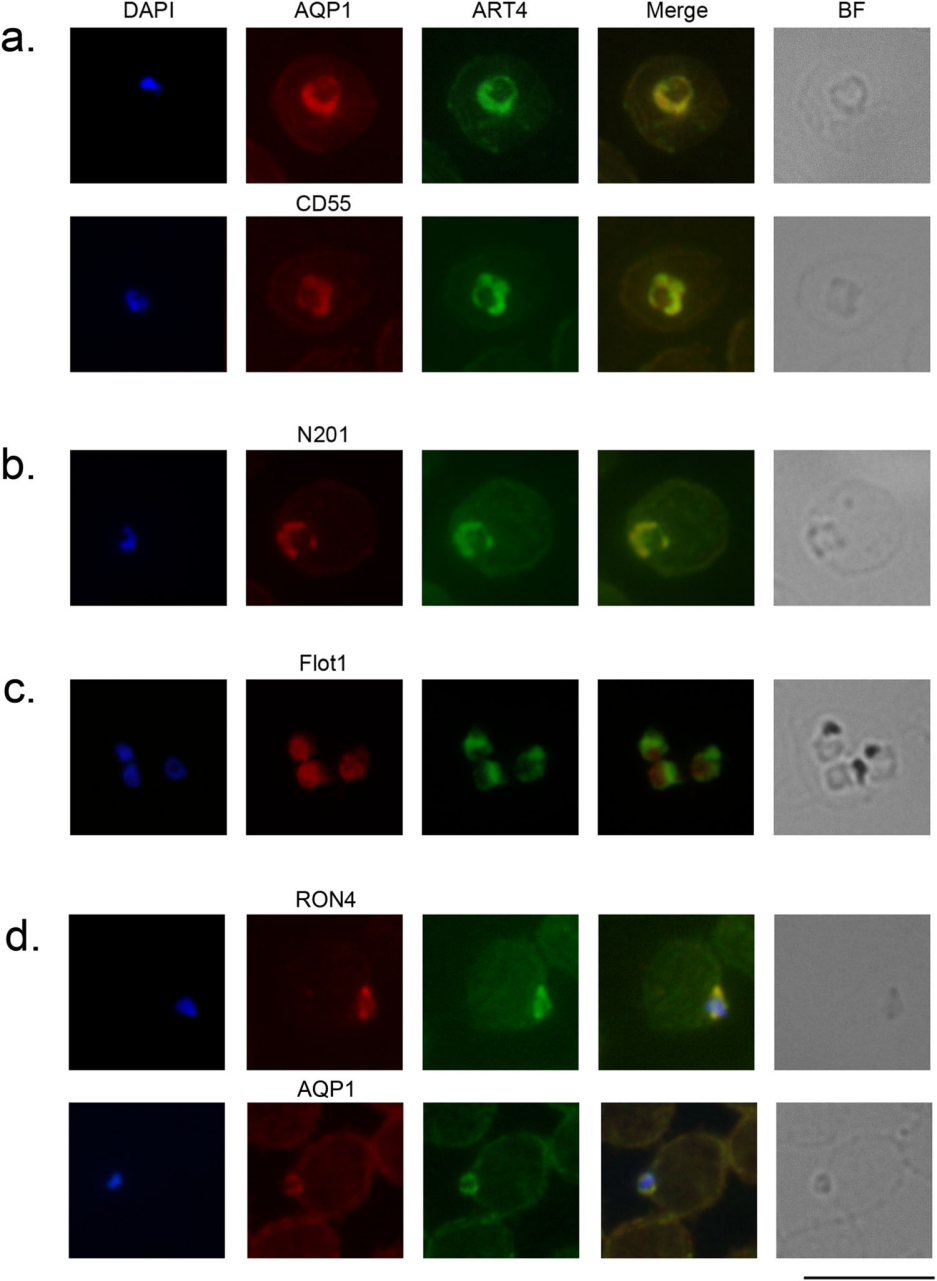
Subcellular localization of CD55, ART4, AQP1, and Flotillin-1. **a** IFA of early trophozoites (ring stages) with anti-ART4, anti-AQP1, and anti-CD55 antibodies. **b** IFA of a ring-stage parasite with anti-ART4 and anti-N201, used as a parasitophorous vacuole marker. **c** IFA of trophozoite stage parasites with visible hemozoin, stained with anti-ART4 and anti-Flotillin-1 (Pearson’s correlation coefficient: 0.56). **d** IFA of invading parasites with anti-ART4 antibody and anti-AQP1 antibody. Anti-RON4 antibody was used as a marker of the moving junction. BF Bright field. Nuclei are stained with DAPI. Scale bar: 10 µm.

Subcellular localization of cluster 3 proteins CD55, ART4, and AQP1 was assessed by immune-fluorescence assay (IFA) with specific antibodies. In non-infected erythrocytes, the three proteins decorate the cell periphery (Supplementary Fig. S4), while in *P. falciparum*-infected erythrocytes they are all internalized by the parasite and co-localize with each other (Fig. 2a). Pearson’s correlation coefficients were 0.97 for AQP1-ART4 and 0.96 for CD55-ART4. Interestingly, other host microdomain proteins reported not to be recruited by *P. falciparum*, such as Band 3, Stomatin, Solute carrier family 2 (Glut1), and Glyceraldehyde-3-phosphate dehydrogenase (GAPDH)^6^ are all grouped in a different cluster (cluster 7).

We also showed that ART4, when internalized, co-localizes with N201 (also referred to as Pf113, PF3D7_1420700), a parasite protein associated with PVM microdomains^26,27^ (Fig. 2b), with a Pearson’s correlation coefficient of 0.96. These results suggest that proteins grouped in cluster 3 are mobilized during *P. falciparum* infection and relocated to the newly formed PVM. This behavior is not common to all internalized proteins; Flotillin-1, grouped in cluster 2, and reported to be internalized during infection^6^, is not associated with the PVM, (Fig. 2c) suggesting that, in this case, internalization occurs via alternative pathways.

To define whether internalization of AQP1 and ART4 occurs during the early stages of invasion, when the PVM is generated, we performed IFAs of parasite cultures enriched in invading merozoites. These experiments showed that, upon invasion, ART4 is recruited to the site of parasite entry, in proximity to the Rhoptry Neck Protein 4 (RON4), a component of the moving junction^28^ (Fig. 2d). We also showed that AQP1 co-localizes with ART4 during merozoite invasion (Fig. 2d). We considered these results suggestive of a possible involvement of the two proteins in the invasion process.

#### Functional analysis: gene knockout studies

**Fig. 3:**
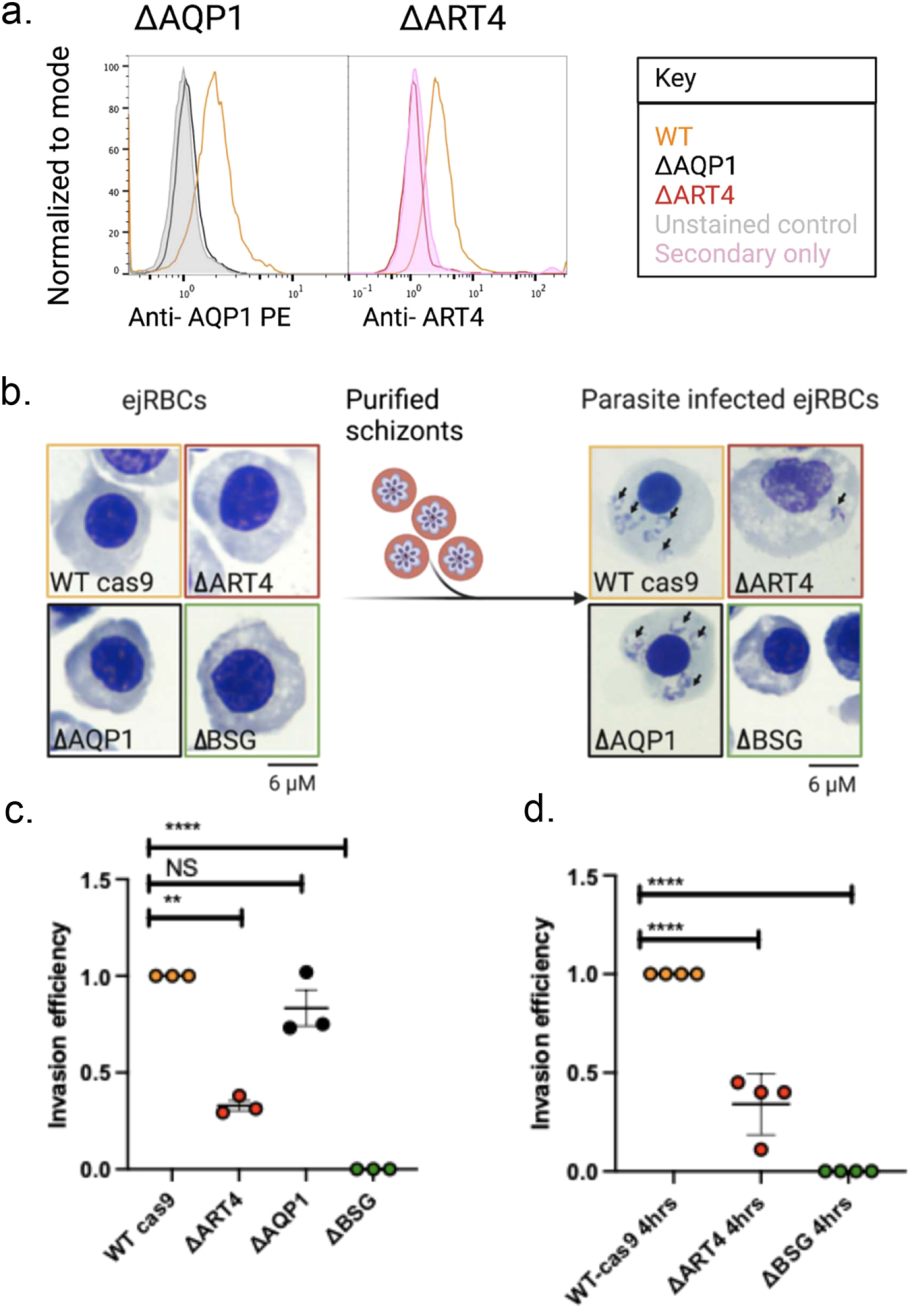
The genetic disruption of ART4 leads to a perturbation in invasion efficiency of *P. falciparum*. **a** Flow plots showing the loss of surface expression of AQP1 and ART4 on their respective ejRBC knockout cells (ΔART4 and ΔAQP1). **b** Representative images of Giemsa-stained cytospins of differentiated day 8 WT cas9, ΔART4, ΔAQP1, and ΔBSG ejRBCs before (left panel) and after (right panel) invasion by *P. falciparum*. Arrows point at intracellular *P. falciparum* parasites. **c** Graph of invasion efficiencies for WT cas9, ΔART4, ΔAQP1, and ΔBSG ejRBCs (*n* = 3) during a 18 h invasion assay. Error bars represent standard deviations. **d** Graph of invasion efficiencies for WT cas9, ΔART4 and ΔBSG ejRBCs (*n* = 3) during a 4 h invasion assay. NS not significant (*p* > 0.05). **p ≤ 0.01, ****p ≤ 0.0001 (one-way ANOVA). ΔBSG was used as a negative control in this experimental assay.

In order to investigate the role of ART4 and AQP1 in *P. falciparum* invasion of the host cell, we generated mutant cell lines with genetic knockouts of either *ART4* or *AQP1* genes in the erythroid EJ cell line^29^. EJ cells can be induced to differentiate to form terminally differentiated orthochromatic/reticulocyte-like cells (ejRBCs) that can support the invasion by *P. falciparum* parasites. We cloned single-guide RNAs (sgRNA) targeting *ART4* and *AQP1* (Supplementary Fig. S5A) into the LentiGuide-Puro vector and virally transduced EJ cells. After 2–4 weeks of selection on puromycin, clonal lines were obtained by limiting dilution. Knockout (KO) of either *ART4* or *AQP1* was confirmed at the genomic level (Supplementary Fig. S5A), resulting in the ΔART4 and ΔAQP1 clonal lines. The absence of surface expression of ART4 and AQP1 in the ko lines was shown by flow cytometry in comparison with WT EJ cells (Fig. 3a). Following induction to form ejRBCs in the ΔART4 and ΔAQP1 clonal lines, both mutant lines demonstrated an early proliferation defect, compared to WT cas9 ejRBCs, followed by normal differentiation (Supplementary Fig. S5B). Cellular morphology and the surface expression levels of proteins that dynamically change through terminal differentiation (CD71, BSG, CD36, CD49d, and CD55), were very similar between the day 8 WT cas9 and ΔART4 and ΔAQP1 ejRBCS (Supplementary Fig. S5C). This result suggests that the genetic disruption of either *ART4* or *AQP1* does not affect the terminal ejRBC development.

To determine the effect of genetic disruption of *ART4* and *AQP1* on parasite invasion, we performed invasion assays with the *P. falciparum* line 3D7 (Fig. 3b). We had previously generated a clonal BSG KO line (ΔBSG) to act as a negative control in this assay. To measure the efficiency of invasion into ΔART4 and ΔAQP1 ejRBCS, we quantified the parasite erythrocyte multiplication rate (PEMR) by light microscopy at 18 h post invasion (Fig. 3c). The genetic disruption of *ART4* resulted in an ~70% reduction in *P. falciparum* invasion efficiency, whereas no significant effect on invasion was observed when *AQP1* was disrupted (Fig. 3b). These data provide functional evidence for ART4, but not AQP1, in *P. falciparum* RBC invasion or intraerythrocytic survival. To further support a role for ART4 in the invasion process versus early intracellular growth, we repeated invasion assays and quantified PEMR at 4 h post invasion (Fig. 3d). We once again observed similar levels of reduced invasion into ΔART4 ejRBCs compared to wild-type cells.

#### Genetic association studies of susceptibility to *P. falciparum* infection and disease

In order to investigate the role of ART4 and AQP1 in susceptibility to malaria natural infection in humans, we have conducted an association study of genetic variation at encoding loci with severe malaria and parasite density in a case–control sample set from Burkina Faso^30,31^, using a candidate gene approach (Supplemental Methods).

**Fig. 4:**
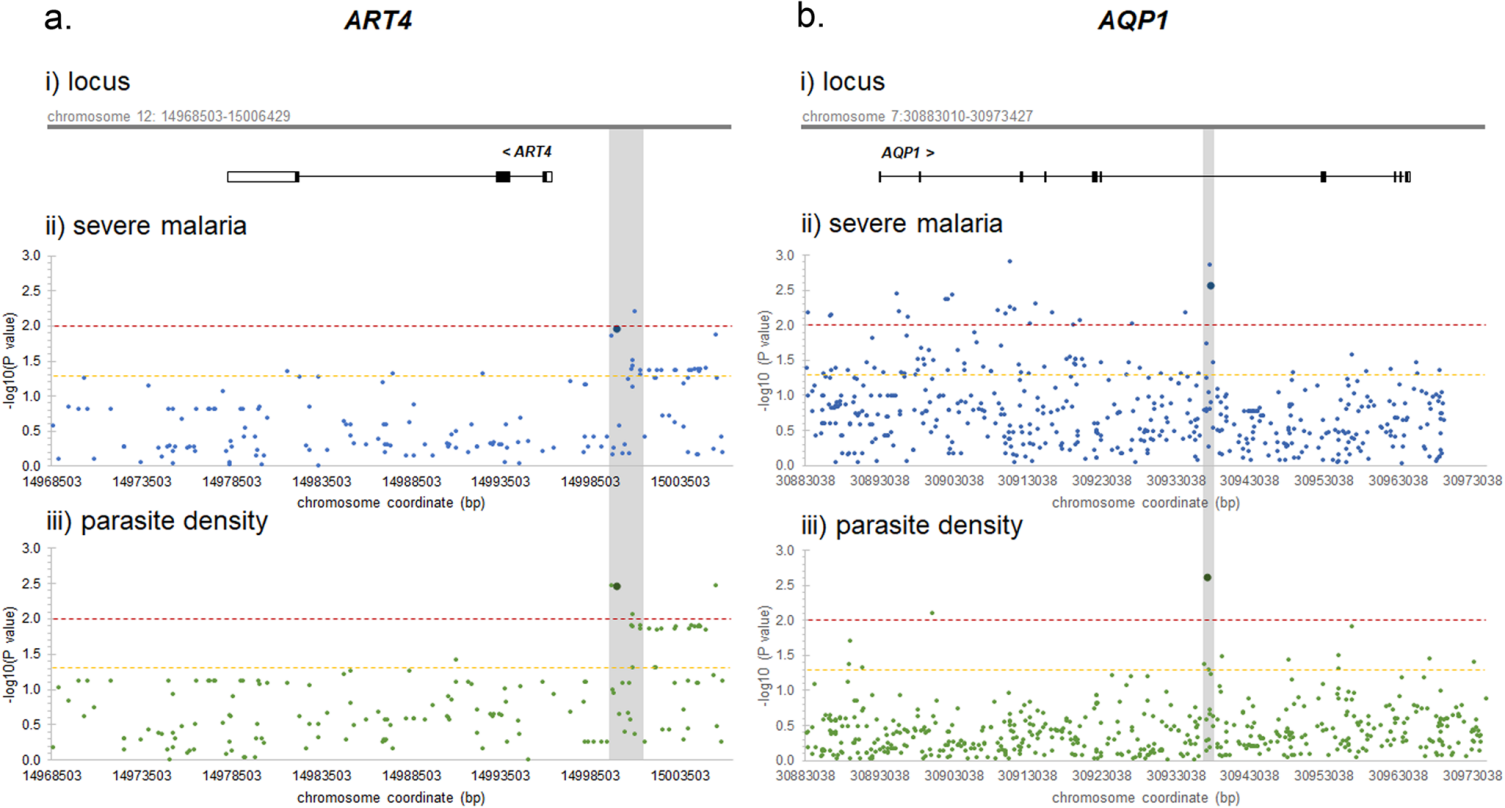
Signals of association between genetic variation at ART4 and AQP1 loci and malaria phenotypes in Mossi children from Burkina Faso. The figure shows results of association analysis of SNPs at *ART4* (**a**) and *AQP1* (**b**) with malaria outcomes in a case–control sample set from Burkina Faso. In panel (i) each locus is shown in its genomic context (chromosome: start base pair-end base pair) with the arrow indicating transcription direction, empty boxes indicating UTR regions, full boxes indicating exons, and lines indicating introns. In panels (ii) and (iii) the *y* axis indicates the minus logarithm (base 10) of the *p*-value for the best model of the likelihood ratio test of association between a given SNP and the malaria phenotype, the *x* axis indicates SNPs ordered by chromosome position, the horizontal dashed lines indicate significance levels (red: *p* = 0.01; yellow: *p* = 0.05), the vertical gray bar indicates the gene region showing significant results with both severe malaria and parasite density, and dots of greater dimension and darker color indicate genotyped SNPs whose results are described in the main text.

A tight cluster of highly significant signals was observed for both phenotypes within a region of ~1.1 kbp (12: 14999719–15000859) corresponding to the upstream of the *ART4* gene (Fig. 4a and Supplementary Data 4), where variants might affect gene expression regulatory elements. The genotyped rs2445411 variant (G/T) (http://grch37.ensembl.org/Homo_sapiens/Variation/Explore?r=12:14999463-15000463;v=rs2445411;vdb=variation;vf=18594244), showing significant association under a dominant model with both severe malaria (OR = 0.64, 95% CI = 0.45–0.90, *p*-value = 0.010) and parasite density (OR = 0.75, 95% CI = 0.62–0.91, *p*-value = 0.003), lies within an open chromatin regulatory feature of 385 base pairs (ENSR00000049171) and affects binding sites for several transcription factors as well as the expression of *ART4* in whole blood and other tissues. It is noteworthy that the T protective allele in 1000 Genomes Project (1000 Genomes Project Consortium 2015) Phase3 data has a frequency of 57% populations of African descent, and of 38% in populations of European descent.

Significant signals of associations are also observed in different regions of the *AQP1* gene (Fig. 4b and Supplementary Data 4) and an intronic region of only ~500 bp (7:30937178–30937725) harbors variants showing highly significant associations with both severe malaria and parasite density. In this region, the best signal of association with severe malaria is shown by the genotyped rs6949918 variant (C/T) (http://grch37.ensembl.org/Homo_sapiens/Variation/Explore?r=7:30937154-30938154;v=rs6949918;vdb=variation;vf=10083937) under an additive model (OR = 0.59, 95% CI = 0.41–0.84, *p*-value = 0.003). The T protective allele has a frequency of 12% in populations of African descent, and of 1% in populations of European descent. The best signal of association with parasite density is shown by the genotyped variant rs1000597 (T/C) (http://grch37.ensembl.org/Homo_sapiens/Variation/Explore?r=7:30936678-30937678;v=rs1000597;vdb=variation;vf=8249466) under a dominant model (OR = 0.64, 95% CI = 0.48–0.85, *p*-value = 0.002). This variant affects the expression of *AQP1* in whole blood and other tissues, and has been reported to be associated with nephrolithiasis^32^, with serum levels of creatinine, uric acid, and calcium, and with urine pH level^33^. The C protective allele has a frequency of 25% in populations of African descent, and of 8% in populations of European descent.

## Discussion

This work aims at giving a comprehensive analysis of the composition, organization, and complexity of cholesterol-rich membrane microdomains of human RBCs with a novel approach, which combines quantitative proteomics and bioinformatics. This whole-cell biochemical description of membrane microdomains suggested an unsuspected heterogeneity in their organization, likely due to the distinct composition of lipid environment they are embedded in, consistent with previous observations that detected nano-domains enriched in sphingomyelin, distinct from those enriched in cholesterol^34^. These distinct chemo-physical features may likely favor a coordinated spatial and temporal segregation of raft-associated pathways.

In some cases, members of DRM clusters also share assigned functional annotations, as in the case of cluster 4, where cytoskeletal proteins are grouped, or cluster 3, enriched in proteins involved in signal transduction. These include Calpain 5, Scramblase 4, AQP1, and the Guanine Nucleotide-binding protein G(i) subunit alpha-2, members of protein families potentially implicated in eryptosis^13^. Interestingly, our proteomic analysis also detected receptors of cell death, such as Glycophorin-C and CD95^35^.

We also identified the majority of the proteins known to be involved in *P. falciparum* invasion, corroborating previous reports of an essential role of cholesterol-rich microdomains in the invasion process^3,5^. Among them, we identified BSG, an essential receptor for *P. falciparum* penetration into the host cells^22^, previously reported to be DRM-associated in tissues other than erythrocytes^36,37^.

A second invasion-related receptor, CD55^8^, localizes to cluster 3 and this prompted us to characterize two additional proteins of this cluster, AQP1 and ART4, in relation to *P. falciparum* infection.

In healthy erythrocytes, CD55, ART4, and AQP1 uniformly decorate the erythrocyte periphery. Upon infection, when invading merozoite contacts the host cell surface, the three proteins coalesce in proximity to the penetration site, most likely in response to parasite-dependent stimuli. In the following stages of *P. falciparum* infection, these three proteins are internalized and co-localize with each other to the PVM. It was previously described that certain host DRM proteins are selectively recruited to the nascent PVM, while others are excluded^6^. In our analysis, these excluded proteins fall in cluster 7, supporting the idea that structural organization of DRMs may affect protein differential uptake and confirming that selective recruitment of DRM proteins during *P. falciparum* infection may reflect the existence of multiple microdomains^6^.

Functional analysis of ART4 and AQP1 showed that ART4—but not AQP1—plays a role in parasite invasion, suggesting that host DRM proteins may be exploited by the parasite to support other processes during intraerythrocytic growth. However, it is also possible that the function of AQP1 in *P. falciparum* invasion is not revealed in ejRBCs, which are very young and nucleated RBC precursors^29^ and that AQP1 function may be enhanced in terminally differentiated RBCs.

We showed that *ART4* and *AQP1* polymorphisms exhibit significant associations with severe malaria and parasite density and that protective allele in both loci occur with higher frequency in populations of African descent compared to those of European descent. Although the genome-wide relevance and the causal nature of these associations remain to be established, it is possible to speculate, in light of the functional results, that *ART4* genetic variation might affect parasite invasion of the RBC, while *AQP1* genetic variation might exert its effect on different parasite developmental stages, or may be on different RBC processes related to malaria pathophysiology. For instance, we might speculate that *Plasmodium* takes advantage of AQP1 recruitment to delay programmed host cell death due to the oxidative stress imposed by the parasite^38^, a strategy already reported in other cell systems^39^.

The analysis of membrane microdomain proteins in natural infections may open new perspectives to the study of this membrane compartment in relation to malaria. Notably, association studies with severe malaria showed that a single nucleotide polymorphism in the ATPase Ca^2+^-transporting Plasma Membrane 4 (PMCA4), also identified in this study as DRM-associated, affects the susceptibility towards *Plasmodium* infection^40^.

In conclusion, a deep characterization of host/parasite interplay may open new avenues to malaria control. Parasite strains resistant to currently available drugs periodically emerge, posing a great threat to the global effort to control the disease. An emerging strategy to limit insurgence of drug-resistance is to target the host instead of the pathogen, being host-targeted molecules less prone to generate resistance and potentially effective against different *P. falciparum* strains and different parasite species^41^. Host-targeted drugs have been tested for different infectious diseases^42,43^, including malaria^44^, and in some cases are already in clinical use^45,46^.

More broadly, our proteomic approach, providing a whole-cell organization of membrane microdomains, may be adopted to study the dynamics of raft association in genetic variants or various pathological conditions of the RBC.

## Methods

### Erythrocyte membrane microdomain purification

Blood collected from 7 donors was washed 4 times in RPMI to remove plasma, platelets, and leukocytes. Residual leukocytes were removed by Plasmodipur filters (Europroxima). Erythrocyte purity was assessed by flow cytometry (Gallios Flow cytometer equipped with 3 lasers: 405, 488, 633 nm; Beckman Coulter). Blood samples were stained with anti-CD45-PB (BD bioscience), anti-CD16-FITC (BD bioscience) to identify granulocytes, and anti-CD61-PE (eBioscence), anti-CD41-FITC (eBioscience) to identify platelets. Data were analyzed by using Kaluza Analysis Software (Beckman Coulter).

RBC membranes obtained by hypotonic lysis (5 mM Na-phosphate, 0.5 mM EDTA, pH 8), were suspended in 0.75 ml of MES-buffered saline (25 mM MES, pH 6.5, 0.15 M NaCl) containing 1% Triton X-100 in ice and homogenized with a potter-elvehjem glass homogenizer. Cell lysates were adjusted to 40% sucrose, overlaid with 1.5 ml of 30% and 1.5 ml of 5% sucrose, and subjected to ultracentrifugation in SW60Ti rotor (Beckman Instruments, 210,000×*g*, 18 h at 4 °C). 12 fractions (375 µl each) were collected from the top of the gradient. Proteins associated with Triton-insoluble membranes (floating to light density fractions 2–8) were precipitated as described by Wessel and Flugge^47^ and analyzed by mass spectrometry (Supplemental Methods). Protein identification data are reported in Supplementary data 1.

### Protein quantification and cluster analysis

Protein abundance in each fraction was determined according to the “label-free” Top3 method^48^, based on the average of the three precursor peptides with the highest intensities. When proteins were identified with two unique peptides, a Top2 value was calculated. Abundance profiles of each protein in the different replicates were merged in a single metaprofile and then submitted to hierarchical average linkage clustering by Cluster 3.0^49^ using Pearson’s correlation as a measure of profile similarity. The resulting dendrogram was visualized by using Java Treeview and clusters of correlated profiles (*R* ≥ 0.6 *p* ≤ 0.005) were extracted by manual analysis.

### Parasite cultures and IFAs

Cultures of the 3D7 *P. falciparum* strain were maintained according to Trager and Jensen with slight modifications^50^.

To produce cultures enriched in merozoites, synchronous and mature *P. falciparum* schizonts were treated for 6 h with E64, a compound that inhibits merozoite egress^51^, and then washed by vigorous pipetting, allowing the released merozoites to reinvade for 5 min. The resulting culture, enriched in invading merozoites, was smeared and tested by double IFA. All other IFA.

Blood smears were fixed 30 min in 4% paraformaldehyde/0,015% glutaraldehyde, permeabilized with 0,1% Triton X-100 in PBS and incubated for 1 h in primary antibodies: mouse monoclonal anti-AQP1 (Invitrogen) 1:50; rabbit polyclonal anti-ART4 (AbNova) 1:100; mouse polyclonal N201 (mouse serum) 1:50^26^. After washing in PBS, smears were incubated in secondary antibodies: anti-mouse rhodamine (Invitrogen) 1:200, anti-rabbit fluorescein (ThermoFisher) 1:200, and with the nuclear marker DAPI (Life Technologies) 500 nG/ml. Smears were mounted in Vectashield™ (Vector Laboratories) and acquired at the fluorescent microscope by using the LAS (v 3.8, Leica Microsystems) software. At least 200 cells were observed in each IFA. Negative controls without primary antibodies were performed.

For co-localization analyses, images of different parasite stages from non-synchronous cultures were acquired by confocal microscope (Olympus) by using the C1-LCSI EZ-C1 software (Olympus) and elaborated by using the plug-in Coloc2 of the program ImageJ.

### Maintenance and differentiation of EJ cells

During both maintenance and differentiation, EJ cells were cultured as described in Scully et al. ^29^. Briefly, maintenance media was comprised of pIMDM supplemented with 5% solvent/detergent virus-inactivated plasma (octaplasma; Octapharma), 3 IU/ml erythropoietin (EPO; Roche), 50 ng/ml stem cell factor (SCF; R&D systems), 1 ng/ml recombinant human IL-3 protein (IL-3; R&D systems), 1 mg/ml doxycycline (Sigma-Aldrich), and 1 mM dexamethasone (Sigma-Aldrich). EJ cells were maintained at a concentration of 50,000 cells/ml, changing media every 2 days.

To induce differentiation, the culture was enriched for basophilic erythroblasts via a 40% (v/v) Percoll-PBS density gradient, seeded at 200,000 cells/ml and cultured for 3 days in pIMDM supplemented with 5% octaplasma, 3 IU/ml EPO, 10 ng/ml SCF, 0.5 ng/ml IL-3, and 0.5 mg/ml doxycycline. On day 3, EJ cells were counted and suspended at 1,000,000 cells/ml in pIMDM containing 5% octaplasma and 3 IU/ml EPO, 5 ng/ml SCF and 0.25 ng/ml Interleukin-3. This cell suspension was seeded onto MS-5 cells that were at 90 % confluency. Terminally differentiated cells were harvested on day 8 of differentiation.

MS-5 cells (DSMZ; ACC 441) were maintained in MEM-alpha (Thermofisher) supplemented with 10% (v/v) heat inactivated fetal bovine serum (FBS) and 0.5% Pen/Strep.

CRISPR/Cas9-mediated genetic perturbations of the EJ cell line were generated via adaptation of previously published protocols^52,53^. We used the lentiCas9-Blast plasmid (Addgene plasmid #52962) and lentiGuide-Puro plasmid (Addgene plasmid # 52963)^52^ to express Cas9 and a single-guide RNA (sgRNA), respectively.

After 2 weeks of selection on 2 μg/ml puromycin, cells expressing the transgene were cloned via limiting dilution, and clones were screened for indels in the gene of interest via Sanger sequencing and TIDE software.

### Flow cytometry

Cells were washed twice in PBS and pelleted in a 96- well plate. For the conjugated antibodies cells were stained in 50 μl of flow buffer (0.1% BSA in 1 × PBS) for 30 min at 4 °C in the dark. Cells were washed twice in flow buffer and resuspended in 100 μl of flow buffer prior to reading. We used the following antibodies at the dilutions indicated by the manufacturer: anti-CD49d PE-Violet 770 (Miltenyi Biotec), anti-CD36 Violet Blue (Miltenyi Biotec), anti-CD71 APC (Miltenyi Biotec), anti-basigin FITC (Invitrogen), anti-CD44 APC (Miltenyi Biotec), anti-CD55 APC (Miltenyi Biotec). Anti-AQP1 PE (B-11; Santa Cruz Biotechnology) was used at a 1:100 dilution. To measure ART4 expression, cells were stained with anti-DO3 eluate at a 1:2 dilution for 30 min at 4 °C in the dark and recognized with anti-human IgG Alexa Fluor 647 (life technologies) at a 1:200 dilution for 1 h 4 °C in the dark. Cells were analyzed by using a MACSQuant analyzer 10 flow cytometer (Miltenyi Biotec) and data were analyzed by using the software FlowJo, version 10.4. 50,000 cells were acquired for each sample and cell populations were separated by a live/dead Propidium iodide stain.

### Invasion assays

3D7 *P. falciparum* cultures were maintained in human O-positive erythrocytes (Interstate Blood bank) at 2% hematocrit in complete RPMI media with 0.5% albumax and 0.2% sodium bicarbonate at 37 °C with 5% CO2 and 1% O_2_ mixed gas.

Invasion assays were performed in the respective parasite maintenance media in 2–3 technical replicates at 0.1% hematocrit and 1% initial parasitemia. We took cytospins immediately after initiation of the invasion assay, as well as 18–20 h or 4 h post invasion. Parasitemia was quantified using a light microscopy, where a minimum of 1000 cells were counted per slide, and presented either as PEMR (parasitized erythrocyte multiplication rate) or invasion efficiency (PEMR normalized to the corresponding control as indicated).

### Genetic association study

The genetic association study was conducted in a malaria case–control sample set from Burkina Faso^30,31^. The study received approval from the ethical committees of the Ministry of Health of Burkina Faso and the University of Oxford. The parents/guardians of children enrolled in the study gave oral informed consent to participation. Clinical data were collected by using validated Case Report Forms, entered into a database independently by two data entry clerks by using EPI Info v6 and checked for accuracy and completeness by using the same software.

DNA samples from children with severe malaria, children with uncomplicated malaria illness and healthy children of Mossi ethnicity aged 0–180 months, of both sexes, were genotyped using the Illumina Omni 2.5 SNP-chip. DNA samples from a reference population of unrelated Mossi subjects were sequenced by Illumina HiSeq 2500, and haplotypes have been used for genotype imputation at unobserved positions. SNP-chip genotyping and Next Generation Sequencing were performed as part of the efforts of the Malaria Genomic Epidemiology Network (www.malariagen.net). Single nucleotide polymorphisms (SNPs) in regions including 10 kb before/after the start/end of each candidate gene (genome assembly CRCh37, Ensemble release 75) were included in the analysis (Supplemental Methods).

### Statistics and reproducibility

For co-localization analyses in IFA, two biological replicates were taken into account and at least 30 cells were analyzed in each replicate. Correlations were assessed by Pearson’s correlation coefficient (*R* ≥ 0.9).

For the functional analysis, three biological replicates were performed, each carried out in triplicate. A minimum of 30 parasites was counted in each experiment, leading to *p*-values probabilities of *p* ≤ 0.01 (one-way ANOVA).

For genetic association studies, DNA samples were collected from children with severe malaria (*n* = 337), children with uncomplicated malaria illness (*n* = 397), and healthy children (*n* = 597) of Mossi ethnicity. DNA samples were also collected from a reference population of 57 unrelated Mossi subjects. Sample size was not pre-determined based on statistical calculation, but was based on the availability of collected clinical data/biological specimens and on pre-established quality control filters.

DRM proteomic analysis was performed on six independent RBC samples prepared from the pooled blood of 7 healthy donors. DRM proteins identified with 1 unique peptide, as well as proteins identified in less than 3 out of 6 replicates, were excluded from the analysis. PAPs were generated for each protein in each replicate and their reproducibility was evaluated by Pearson’s correlation coefficient (*R* ≥ 0.6, *p* < 0.005).

The wild-type and CRISPR knockout erythroid cell lines were differentiated three independent times. From these independent differentiation experiments, invasion assays were set up in triplicate. Cytospins taken at 0 and 20 h post invasion were counted blind by light microscopy.

### Reporting summary

Further information on research design is available in the Nature Research Reporting Summary linked to this article.

## Supplementary information

Supplementary Information

Supplementary Data 1

Supplementary Data 2

Supplementary Data 3

Supplementary Data 4

Reporting Summary

## Data Availability

Raw mass spectrometry data have been deposited in MassIVE (https://massive.ucsd.edu/ProteoSAFe/static/massive.jsp). Username: MSV000086413; Password 3ryMM. As part of the Malaria Genomic Epidemiology Network, data used for genetic association analysis are available as follows: Illumina Omni 2.5M genotype data from study samples have been deposited in the European Genome-Phenome Archive (EGA; study accession EGAS00001001311); whole-genome sequence read data have been deposited in the EGA (study accession EGAS00001003648); access to MalariaGEN datasets on EGA is by application to an independent data access committee. All other data are available from the corresponding author on reasonable request.

## References

[1] Lingwood D, Simons K (2010). Lipid rafts as a membrane-organizing principle. Science.

[2] Bagam P, Singh DP, Inda ME, Batra S (2017). Unraveling the role of membrane microdomains during microbial infections. Cell Biol. Toxicol..

[3] Samuel BU (2001). The role of cholesterol and glycosylphosphatidylinositol-anchored proteins of erythrocyte rafts in regulating raft protein content and malarial infection. J. Biol. Chem..

[4] World Health Organization. World Malaria Report 2019. (2019).

[5] Koshino I, Takakuwa Y (2009). Disruption of lipid rafts by lidocaine inhibits erythrocyte invasion by *Plasmodium falciparum*. Exp. Parasitol..

[6] Murphy SC (2004). Erythrocyte detergent-resistant membrane proteins: their characterization and selective uptake during malarial infection. Blood.

[7] Fratini F (2017). An integrated approach to explore composition and dynamics of cholesterol-rich membrane microdomains in sexual stages of malaria parasite. Mol. Cell Proteomics.

[8] Egan ES (2015). Malaria. A forward genetic screen identifies erythrocyte CD55 as essential for *Plasmodium falciparum* invasion. Science.

[9] Bryk AH, Wiśniewski JR (2017). Quantitative analysis of human red blood cell proteome. J Proteome Res..

[10] Ravenhill BJ (2019). Quantitative comparative analysis of human erythrocyte surface proteins between individuals from two genetically distinct populations. Commun. Biol..

[11] Bharadwaj A, Bydoun M, Holloway R, Waisman D (2013). Annexin A2 heterotetramer: structure and function. Int. J. Mol. Sci..

[12] Aranda JF (2011). MYADM regulates Rac1 targeting to ordered membranes required for cell spreading and migration. Mol. Biol. Cell.

[13] Föller M, Huber SM, Lang F (2008). Erythrocyte programmed cell death. IUBMB Life.

[14] Ciana A, Achilli C, Balduini C, Minetti G (2011). On the association of lipid rafts to the spectrin skeleton in human erythrocytes. Biochim. Biophys. Acta.

[15] Trybus M, Niemiec L, Biernatowska A, Hryniewicz-Jankowska A, Sikorski AF (2019). MPP1-based mechanism of resting state raft organization in the plasma membrane. Is it a general or specialized mechanism in erythroid cells?. Folia Histochem. Cytobiol..

[16] Salzer U, Prohaska R (2001). Stomatin, flotillin-1, and flotillin-2 are major integral proteins of erythrocyte lipid rafts. Blood.

[17] Rungaldier S, Oberwagner W, Salzer U, Csaszar E, Prohaska R (2013). Stomatin interacts with GLUT1/SLC2A1, band 3/SLC4A1, and aquaporin-1 in human erythrocyte membrane domains. Biochim Biophys. Acta.

[18] Salzer U, Hinterdorfer P, Hunger U, Borken C, Prohaska R (2002). Ca(++)-dependent vesicle release from erythrocytes involves stomatin-specific lipid rafts, synexin (annexin VII), and sorcin. Blood.

[19] Bartholdson SJ (2012). Semaphorin-7A is an erythrocyte receptor for *P. falciparum* merozoite-specific TRAP homolog, MTRAP. PLoS Pathog..

[20] Harrison T (2003). Erythrocyte G protein-coupled receptor signaling in malarial infection. Science.

[21] Jaskiewicz E, Jodłowska M, Kaczmarek R, Zerka A (2019). Erythrocyte glycophorins as receptors for *Plasmodium* merozoites. Parasit. Vectors.

[22] Crosnier, C. et al. Basigin is a receptor essential for erythrocyte invasion by *Plasmodium falciparum*. *Nature* **480**, 534–537 (2011). 10.1038/nature10606 PMC3245779 22080952

[23] Egan ES (2018). Erythrocytes lacking the Langereis blood group protein ABCB6 are resistant to the malaria parasite *Plasmodium falciparum*. Commun. Biol..

[24] Paone S (2020). Characterization of the erythrocyte GTPase Rac1 in relation to *Plasmodium falciparum* invasion. Sci. Rep..

[25] de Souza Santos M, Orth K (2015). Subversion of the cytoskeleton by intracellular bacteria: lessons from *Listeria*, *Salmonella* and *Vibrio*. Cell Microbiol..

[26] Yam XY (2013). Proteomic analysis of detergent-resistant membrane microdomains in trophozoite blood stage of the human malaria parasite *Plasmodium falciparum*. Mol Cell Proteomics.

[27] Sanders PR (2005). Distinct protein classes including novel merozoite surface antigens in Raft-like membranes of *Plasmodium falciparum*. J. Biol. Chem..

[28] Alexander DL, Arastu-Kapur S, Dubremetz JF, Boothroyd JC (2006). *Plasmodium falciparum* AMA1 binds a rhoptry neck protein homologous to TgRON4, a component of the moving junction in *Toxoplasma gondii*. Eukaryot. Cell.

[29] Scully EJ (2019). Generation of an immortalized erythroid progenitor cell line from peripheral blood: a model system for the functional analysis of *Plasmodium* spp. invasion. Am. J. Hematol..

[30] Modiano D (1998). Severe malaria in Burkina Faso: influence of age and transmission level on clinical presentation. Am. J. Trop. Med. Hyg..

[31] Modiano D (2001). Haemoglobin C protects against clinical *Plasmodium falciparum* malaria. Nature.

[32] Urabe Y (2012). A genome-wide association study of nephrolithiasis in the Japanese population identifies novel susceptible Loci at 5q35.3, 7p14.3, and 13q14.1. PLoS Genet..

[33] Wang L (2017). Association study of reported significant loci at 5q35.3, 7p14.3, 13q14.1 and 16p12.3 with urolithiasis in Chinese Han ethnicity. Sci. Rep..

[34] Leonard C (2017). Contribution of plasma membrane lipid domains to red blood cell (re)shaping. Sci. Rep..

[35] Gajate C, Mollinedo F (2015). Lipid rafts and raft-mediated supramolecular entities in the regulation of CD95 death receptor apoptotic signaling. Apoptosis.

[36] Grass GD, Bratoeva M, Toole BP (2012). Regulation of invadopodia formation and activity by CD147. J. Cell Sci..

[37] Miranda PV, Allaire A, Sosnik J, Visconti PE (2009). Localization of low-density detergent-resistant membrane proteins in intact and acrosome-reacted mouse sperm. Biol. Reprod..

[38] Boulet C, Doerig CD, Carvalho TG (2018). Manipulating eryptosis of human red blood cells: a novel antimalarial strategy?. Front. Cell Infect. Microbiol..

[39] Jablonski EM (2007). Decreased aquaporin expression leads to increased resistance to apoptosis in hepatocellular carcinoma. Cancer Lett..

[40] Timmann C (2012). Genome-wide association study indicates two novel resistance loci for severe malaria. Nature.

[41] Chiang, C. Y. et al. Mitigating the impact of antibacterial drug resistance through host-directed therapies: current progress, outlook, and challenges. *mBio* **9**, 10.1128/mBio.01932-17 (2018). 10.1128/mBio.01932-17 PMC5790911 29382729

[42] Stanley SA (2014). Identification of host-targeted small molecules that restrict intracellular *Mycobacterium tuberculosis* growth. PLoS Pathog..

[43] de Wispelaere M, LaCroix AJ, Yang PL (2013). The small molecules AZD0530 and dasatinib inhibit dengue virus RNA replication via Fyn kinase. J. Virol..

[44] Brizuela M (2014). Treatment of erythrocytes with the 2-cys peroxiredoxin inhibitor, Conoidin A, prevents the growth of Plasmodium falciparum and enhances parasite sensitivity to chloroquine. PLoS ONE.

[45] Latinovic O, Kuruppu J, Davis C, Le N, Heredia A (2009). Pharmacotherapy of HIV-1 Infection: focus on CCR5 antagonist maraviroc. Clin. Med. Ther..

[46] Crouchet E, Wrensch F, Schuster C, Zeisel MB, Baumert TF (2018). Host-targeting therapies for hepatitis C virus infection: current developments and future applications. Therap. Adv. Gastroenterol..

[47] Wessel D, Flügge UI (1984). A method for the quantitative recovery of protein in dilute solution in the presence of detergents and lipids. Anal. Biochem..

[48] Silva JC, Gorenstein MV, Li GZ, Vissers JP, Geromanos SJ (2006). Absolute quantification of proteins by LCMSE: a virtue of parallel MS acquisition. Mol. Cell Proteomics.

[49] de Hoon MJ, Imoto S, Nolan J, Miyano S (2004). Open source clustering software. Bioinformatics.

[50] Trager W, Jensen JB (1976). Human malaria parasites in continuous culture. Science.

[51] Salmon BL, Oksman A, Goldberg DE (2001). Malaria parasite exit from the host erythrocyte: a two-step process requiring extraerythrocytic proteolysis. Proc. Natl Acad. Sci. USA.

[52] Sanjana NE, Shalem O, Zhang F (2014). Improved vectors and genome-wide libraries for CRISPR screening. Nat. Methods.

[53] Shalem O (2014). Genome-scale CRISPR-Cas9 knockout screening in human cells. Science.

