# Supplementary material for: Structural organization of erythrocyte membrane microdomains and their relation with malaria susceptibility
Source: Commun Biol. 2021 Dec 8;4:1375. doi: 10.1038/s42003-021-02900-w (PMC8655059; doi:10.1038/s42003-021-02900-w)
Supplement: Supplementary file 1 — Supplementary Information [file 42003_2021_2900_MOESM1_ESM.pdf]

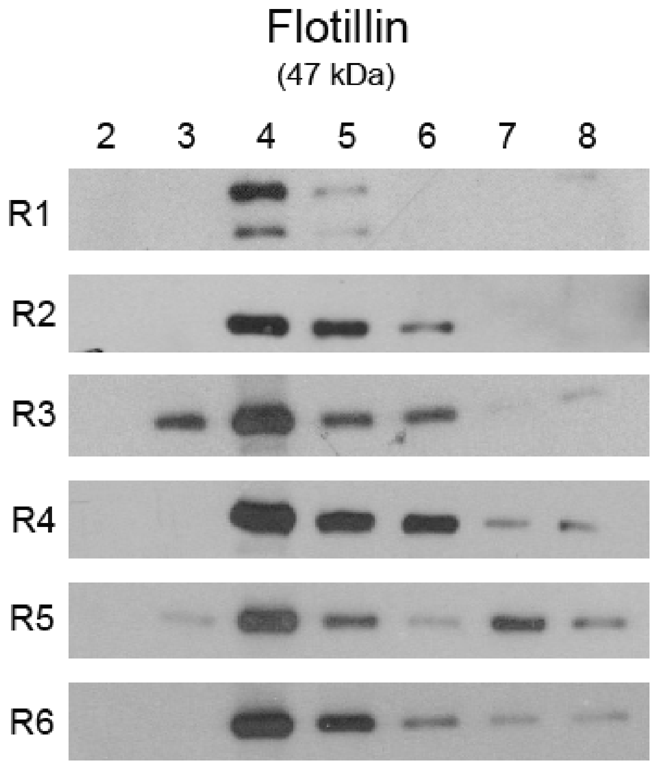

Figure S1. Western blot analysis of DRMs used in proteomic analysis. Sucrose gradient light fractions (2-8) were probed with antibodies specific for Flotillin 1. In all replicates (R1-R6) used in proteomics, Flotillin 1 peaks at fraction 4.

A

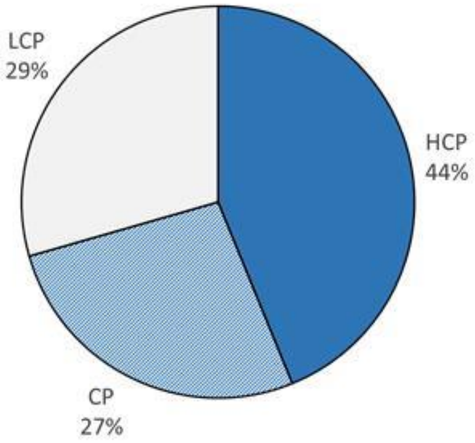

B

Cluster	HCP	CP	LCP
c1	0.0	0.0	100.0
c2	80.0	20.0	0.0
c3	15.4	53.8	30.8
c4	46.2	15.4	38.5
c5	100.0	0.0	0.0
c6	52.0	24.0	24.0
c7	38.5	34.6	26.9
c8	65.0	35.0	0.0
c9	0.0	33.3	66.7
nc	45.0	25.0	30.0

C

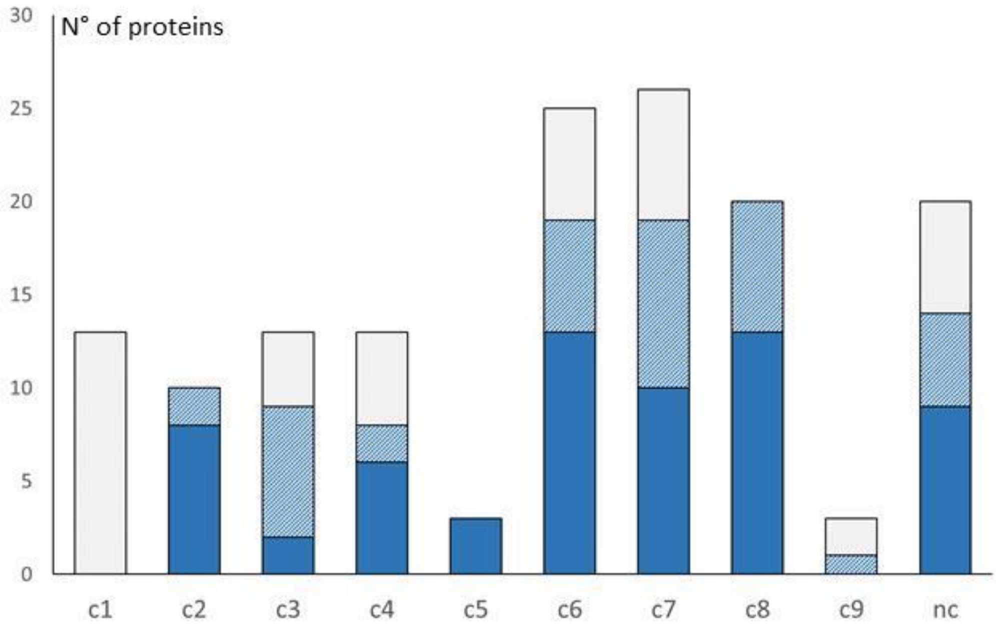

Figure S2. Reproducibility of protein abundance profiles (PAPs). PAPs identified in at least three replicates were defines as highly conserved (HCP) when having an R ≥0.6 ($p < 0.005$) in more than 70% of compared profile pairs; conserved (CP) when having an R≥0.6 in 50-70% profile pairs and low conserved (LCP) when having an R≥0.6 in less than 50% profile pairs. A) PAP reproducibility is presented as a pie chart showing that about 70% of proteins have conserved profiles (HCP or CP). B) the percentage of conserved PAPs varies between clusters (c) and non-clusterized proteins (nc). C) Distribution of PAP reproducibility in each cluster is shown as a stacked bar chart.

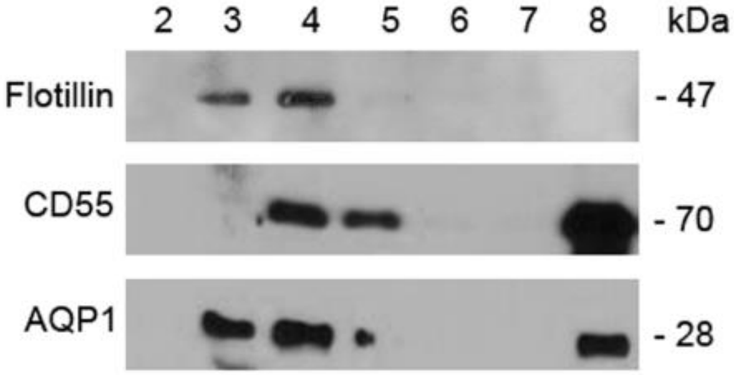

Figure S3. Ghost DRM analysis from a second pool of donors. Sucrose gradient light fractions (2-8) were probed with antibodies specific for Flotillin 1, CD55 and AQP1. Western blot analysis shows that Flotillin 1 peaks to fraction 4, while CD55 and AQP1 to fraction 4 and 8 as in the case of western blot and/or PAP analysis of the first pool of donors.

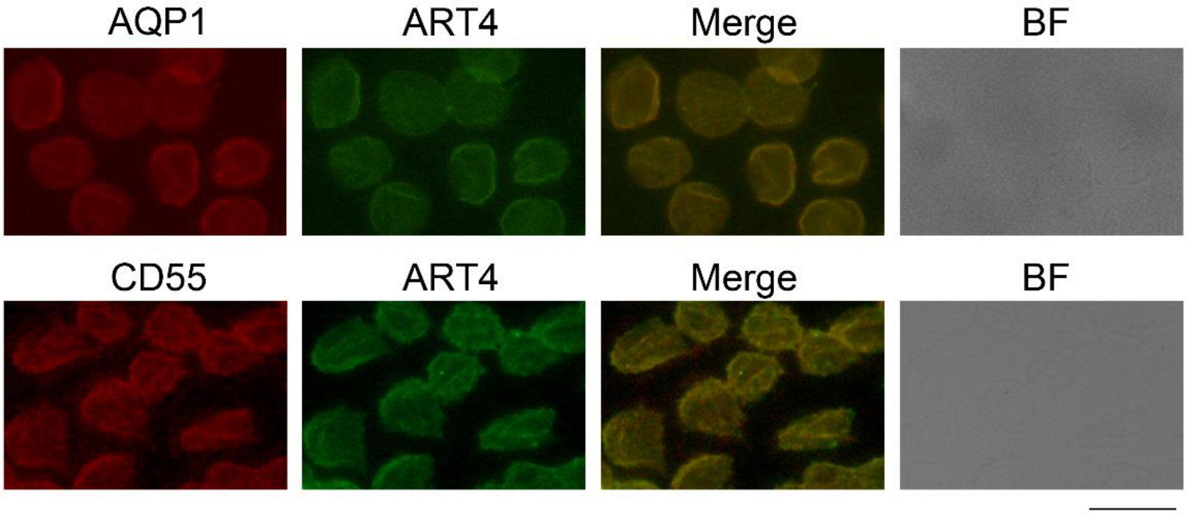

Figure S4. AQP1, ART4 and CD55 localization in non-infected erythrocytes. IFAs of uninfected human erythrocytes with anti-AQP1, anti-ART4 and anti-CD55. BF: bright field. Scale bar: 10 µm.

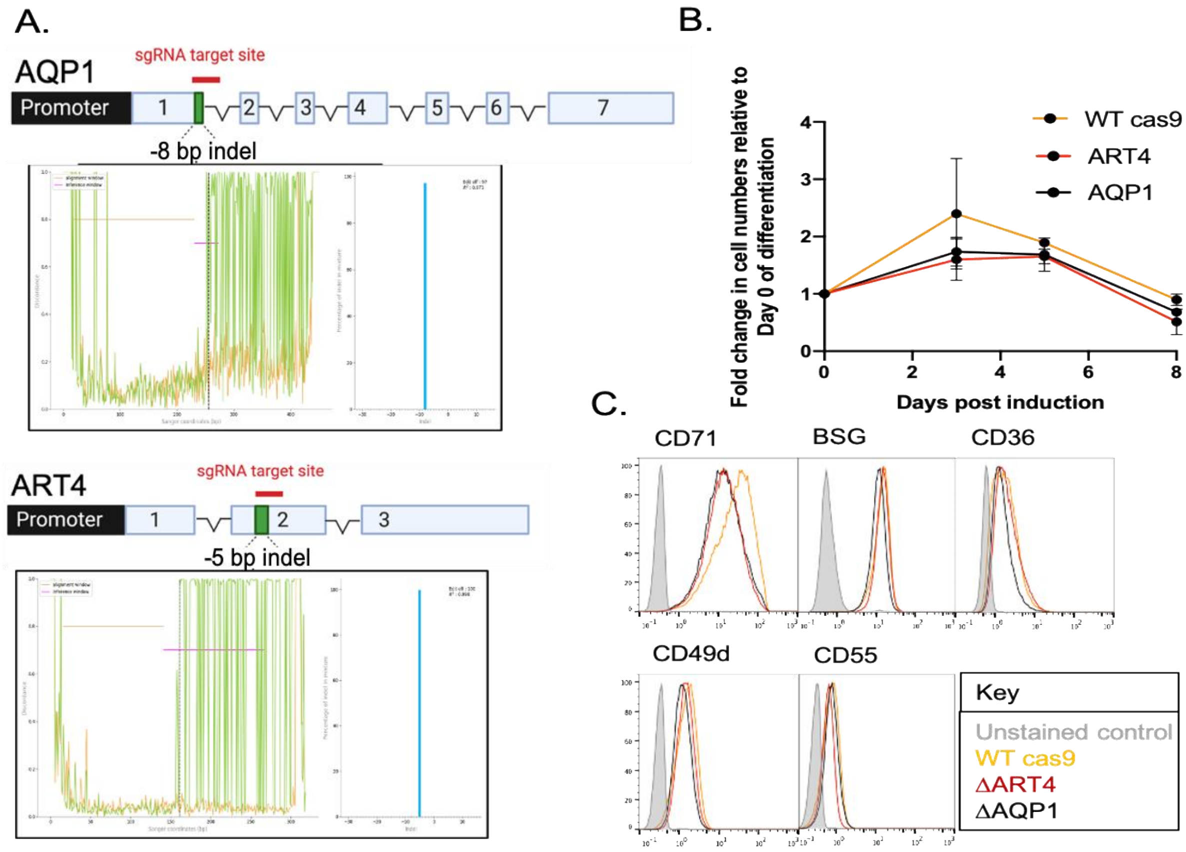

Figure S5. The genetic disruption of *ART4* does not alter terminal differentiation. A. The sgRNA guide targets a PAM site in exon 2 of *ART4* at bases 3186-8. The sgRNA guide targets a PAM site in exon 1 of *AQP1* at bases 1192-4. The boxes show TIDE analysis results, indicating the spectrum and frequencies of indels present in each gene. B. Growth dynamics of WT cas9, ΔART4 and ΔAQP1 EJ cells across differentiation. C. Flow plots showing surface expression of host receptors and additional markers whose abundance is associated with maturation.

Supplemental Methods

Proteomic analysis

<u>*Sample preparation*</u>. Proteins from collected DRM fractions 2-8 were loaded on SDS-PAGE (home-made 5% stacking-12% resolving bis-tris-PAGE) and run just to allow the Protein Marker (BenchMark™ Pre-Stained Protein Ladder, Invitrogen) to enter the resolving gel. After Coomassie Staining (Novex, Colloidal Blue Staining gel, Invitrogen) unresolved bands were excised and in-gel tryptic digestion was performed as

previously described[1]. Briefly, gel slices were destained by washings in acetonitrile (ACN)/50 mM NH4CO3 (1:1), treated with 10 mM DTT (40 min at 56°C) and 55 mM iodoacetamide (30 min in the dark at RT) to reduce and alkylate cysteines, shrunken with ACN and rehydrated for 40 min on ice with a solution of 12,5 ng/µl trypsin (Promega) in 50 mM NH4CO3 and protein digestion was carried out overnight at 37°C. Trypsin digests were removed and gel pieces were shrank with 95% ACN, 1% FA. Supernatants were combined with trypsin digests, the volume reduced in speedvac and adjusted to 20µl with HPLC buffer A (95% ACN, 0.1% FA).

Liquid Chromatography Tandem Mass Spectrometry. Nano-RPLC was performed using a nano-HPLC 3000 Ultimate (Dionex) connected in line to LTQ-XL linear ion trap (Thermo Fisher). Tryptic digests were first loaded on a C18 RP-precolumn (300µm i.d.x5 mm; 5µm particle size; 100 Å pore size; LC Packings-Dionex), washed by loading pump at 20 µl/min with buffer A for 5 min and then on a home-made 13 cm x 75 µm- i.d. Silica PicoTip (8 ±1µm) column (PicoTip Emitter, NewObjective) packed with Magic C18AQ (5µm particle size; 200 Å pore size, Michrom Bioresouces Inc.) for chromatographic separations. Peptides were eluted at 0.3 µL/min along a 120 min linear gradient from 20% to 60% of buffer B (95% ACN, 0.1% FA) and electrosprayed directly into the mass spectrometer. Data acquisition was performed in data-dependent Top5. Full-scan MS was set with a maximum injection time of 10 ms and m/z 400-2000 mass range. The five most intense ions were sequentially selected and fragmented in CID mode: maximum injection time of 100 ms; m/z 50-2000 mass range; minimum signal threshold of 100 counts. Wide band activation and dynamic exclusion were enabled.

Mass spectrometry data processing. Spectra files were analyzed by Sequest HT search engine with Proteome Discoverer 1.4 (ThermoFisher) using the UniProtKB/Swiss-Prot Human reviewed database released in 2020. The Carboamidomethylation of cysteines was specified as fixed modification while the oxidation of methionine and phosphorylation of Serine, Threonine and Tyrosine were set as variable modification; only full tryptic peptides were used for identification, mass tolerance was set to 1 Da for precursor ion and 0.4 Da for fragment ions and a maximum of two missed cleavages was allowed. The Percolator tool was used for peptide validation based on the q-value and high confidence was chosen, corresponding to a false discovery rate (FDR) ≤1% on protein and peptide-level. Proteins were identified with a minimum of 2 peptides rank=1, while maximum peptide rank was 2. Score versus Charge State was set as following: Minimal Score for charge state

= 1: 1.5, Minimal Score for charge state = 2: 2, Minimal Score for charge state = 3: 2.5, Minimal Score for charge state = 4: 3. For peptides and proteins grouping default settings were used.

Genetic association analysis

Sample recruitment

The sample of severe malaria cases was recruited at the 158-bed paediatric ward of the Ouagadougou University Hospital. In line with WHO guidelines, severe malaria was defined by the presence of *P. falciparum* in the thick blood film associated with at least one of the following conditions: prostration (incapacity of the child to sit without help in the absence of coma), unrousable coma (score between 0 and 2 on the Glasgow modified coma scale), repeated generalised convulsions (more than two episodes in the preceding 24 h), severe anaemia (haemoglobin <5 g per 100 ml), hypoglycaemia (<40 mg per 100 ml), pulmonary oedema/respiratory distress, spontaneous bleeding and renal failure (plasma creatinine>3 mg per 100 ml). The sample of healthy control children was recruited during malaria cross-sectional surveys performed in the Ouagadougou area. The same case-control sample set was used in previously published studies that could demonstrate significant associations of a range of effect sizes [2-4].

Association studies

Quality control filtering of variants has been performed using QCtoolv2 (options: -hwe 5, -maf 0.01 0.99, -snp-missing-rate 0.10, -snp-missing-call-rate 0.9). Imputation with haplotype panels from Mossi and 1000Genomes populations has been performed using IMPUTEv2 by MCMC algorithm (options: -buffer 500, -k 100, -k_hap 100), with a resulting concordant rate>95%. Association testing of SNP genotypes (AA, AB, BB; where A is the reference allele and B is the alternative allele) with severe malaria has been performed using SNPTESTv2.5.2 by likelihood ratio test under additive (AA vs AB vs BB, assuming each additional copy of the alternative allele increases risk by the same amount), dominant (AA+AB vs BB), recessive (AA vs AB+BB), general (AA vs AB vs BB, with no assumptions) and heterozygote (AB vs AA+BB) models (options: -frequentist 1to5, -method newml) [5]. Association testing with parasite density (log-normalized number of parasites per microliter of blood) has been performed among all malaria cases (n = 734) using SNPTESTv2.5.2 by likelihood ratio test on linear regression under additive, dominant, recessive, general and

heterozygote models (options: -frequentist 1to5, -method expected)[5]. The *HBB* gene has been analyzed along with candidate genes to serve as a positive control. Association results of the rs334 (A/T) variant, where the alternative allele encodes haemoglobin S, with severe malaria and parasite density, are, respectively: OR=0.12, 95%CI=0.05-0.30, p-value=$9x10^{-6}$; OR=0.74, 95%CI=0.54-1.01, p-value=0.056.

Supplementary References:

1. Fratini F, Raggi C, Sferra G, et al. An Integrated Approach to Explore Composition and Dynamics of Cholesterol-rich Membrane Microdomains in Sexual Stages of Malaria Parasite. *Mol Cell Proteomics*. 2017;16(10):1801-1814.
2. Modiano D, Luoni G, Sirima B, et al. Haemoglobin C protects against clinical Plasmodium falciparum malaria. *Nature*. 2001;414(6861):305-308.
3. Mangano VD, Luoni G, Rockett KA, et al. Interferon regulatory factor-1 polymorphisms are associated with the control of Plasmodium falciparum infection. *Genes Immun*. 2008;9(2):122-129.
4. Network MGE. Insights into malaria susceptibility using genome-wide data on 17,000 individuals from Africa, Asia and Oceania. *Nat Commun*. 2019;10(1):5732.
5. Howie B, Marchini J, Stephens M. Genotype imputation with thousands of genomes. *G3 (Bethesda)*. 2011;1(6):457-470.
