# Supplementary material for: Structural organization of erythrocyte membrane microdomains and their relation with malaria susceptibility
Source: Commun Biol. 2021 Dec 8;4:1375. doi: 10.1038/s42003-021-02900-w (PMC8655059; doi:10.1038/s42003-021-02900-w)
Supplement: Supplementary file 6 — Reporting Summary [file 42003_2021_2900_MOESM6_ESM.pdf]

nature research

Corresponding author(s): *Double-blind peer review submissions: write DBPR and your manuscript number here instead of author names.*

Last updated by author(s): *YYYY-MM-DD*

Reporting Summary

Nature Research wishes to improve the reproducibility of the work that we publish. This form provides structure for consistency and transparency in reporting. For further information on Nature Research policies, see our Editorial Policies and the Editorial Policy Checklist.

Statistics

For all statistical analyses, confirm that the following items are present in the figure legend, table legend, main text, or Methods section.

n/a	Confirmed	
☐	☒	The exact sample size (*n*) for each experimental group/condition, given as a discrete number and unit of measurement
☐	☒	A statement on whether measurements were taken from distinct samples or whether the same sample was measured repeatedly
☐	☒	The statistical test(s) used AND whether they are one- or two-sided<br>*Only common tests should be described solely by name; describe more complex techniques in the Methods section.*
☒	☐	A description of all covariates tested
☒	☐	A description of any assumptions or corrections, such as tests of normality and adjustment for multiple comparisons
☐	☒	A full description of the statistical parameters including central tendency (e.g. means) or other basic estimates (e.g. regression coefficient) AND variation (e.g. standard deviation) or associated estimates of uncertainty (e.g. confidence intervals)
☐	☒	For null hypothesis testing, the test statistic (e.g. F, t, r) with confidence intervals, effect sizes, degrees of freedom and P value noted<br>*Give P values as exact values whenever suitable.*
☒	☐	For Bayesian analysis, information on the choice of priors and Markov chain Monte Carlo settings
☒	☐	For hierarchical and complex designs, identification of the appropriate level for tests and full reporting of outcomes
☐	☒	Estimates of effect sizes (e.g. Cohen's *d*, Pearson's *r*), indicating how they were calculated

Our web collection on statistics for biologists contains articles on many of the points above.

Software and code

Policy information about availability of computer code

Data collection	C1-LCSI EZ-C1 software by Olympus, Tokyo, Japan (confocal microscope), LAS (v 3.8, Leica Microsystems) software (fluorescence microscope). For genetic analysis, clinical data were collected using validated Case Report Forms, entered into a database independently by two data entry clerks using EPI Info v6, checked for accuracy and completeness using the same software.
Data analysis	Cluster3.0 and JavaTreeview (for cluster analysis and heatmap/dendrogram visualization), Coloc2, plugin of the software ImageJ (for co-localization analysis), FlowJo, version 10.4 (FACS analysis of erythroid markers on EJ cells,), Kaluza software v.2.0 (Beckman Coulter) (FACS analysis of mature erythrocytes for proteomics).<br>For genetic analysis, the following softwares were used: QCtoolv2 for quality control, IMPUTEv2 for genotype imputation, SNPTESTv2.5.2 for association testing. Softwares download and documentation are available at www.malariagen.net/resource/25.

For manuscripts utilizing custom algorithms or software that are central to the research but not yet described in published literature, software must be made available to editors and reviewers. We strongly encourage code deposition in a community repository (e.g. GitHub). See the Nature Research guidelines for submitting code & software for further information.

Data

Policy information about availability of data

All manuscripts must include a data availability statement. This statement should provide the following information, where applicable:

- Accession codes, unique identifiers, or web links for publicly available datasets
- A list of figures that have associated raw data
- A description of any restrictions on data availability

Row mass spectrometry data have been deposited in MassIVE (ftp://MSV000086413@massive.ucsd.edu). Username: MSV000086413; Password 3ryMM.
As part of the Malaria Genomic Epidemiology Network, data used for genetic association analysis are available as follows: Illumina Omni 2.5 M genotype data from

study samples have been deposited in the European Genome-Phenome Archive (EGA; study accession EGAS00001001311); whole-genome sequence read data have been deposited in the EGA (study accession EGAS00001003648); access to MalariaGEN datasets on EGA is by application to an independent data access committee.

Field-specific reporting

Please select the one below that is the best fit for your research. If you are not sure, read the appropriate sections before making your selection.

- [x] Life sciences
- [ ] Behavioural & social sciences
- [ ] Ecological, evolutionary & environmental sciences

For a reference copy of the document with all sections, see nature.com/documents/nr-reporting-summary-flat.pdf

Life sciences study design

All studies must disclose on these points even when the disclosure is negative.

Sample size	For co-localization analyses, two biological replicates were taken into account and at least 30 cells were analyzed in each replicate, leading to average Pearson's correlation coefficients of 0.97 for AQP1-ART4, 0.96 for CD55-ART4 and 0.96 for N201-ART4 with standard deviations <0.01.<br>For the functional analysis, 3 biological replicates were performed, each carried out in triplicate. A minimum of 30 parasites were counted in each experiment, leading to P values probabilities (one-way ANOVA) of $P \leq 0.01$ for the wt/ART4 ko comparison and non significance for the wt/AQP1 ko.<br>For genetic association study, sample size was not pre-determined based on statistical calculation, but was based on the availability of collected clinical data/biological specimens and on pre-established quality control filters. The same case-control sample set has been used in previously published studies that could demonstrate significant associations of a range of effect sizes (doi: 10.1038/35104556; doi: 10.1038/sj.gene.6364456; doi: 10.1038/s41467-019-13480-z).
Data exclusions	No data were excluded from the analyses, except for proteomic data.<br>For proteomic analysis, proteins identified with 1 unique peptide were excluded from data analysis (functional annotation, comparative analysis). To improve robustness of the cluster analysis (Pearson's correlation (R) ≥0.6 with a Probability value (P) ≤ 0.005), proteins identified in less than 3 out of 6 replicates were excluded.
Replication	For proteomic data analysis, effectiveness and reproducibility of Detergent Resistant Membrane (DRM) separation was assessed by probing sucrose gradient fractions with an antibody against Flotillin (a DRM marker).<br>To assess reproducibility of the Protein Abundance Profiles (PAPs), the Pearson's correlation was calculated. We verify that more than 70% of PAP pairs are conserved ($R \geq 0.6$, $P<0.005$). The presence of a protein subset with less conserved PAPs was explained by the presence of DRM-associated proteins residing in membrane contexts partially susceptible to detergent extraction. Moreover, PAP reproducibility was confirmed in a different blood sample by an alternative method (Western blot).<br><br>The wild type and CRISPR knock out erythroid cell lines were differentiated three independent times. From these independent differentiation experiments, invasion assays were set up in triplicate. Cytospins taken at 0hrs and 20hrs post invasion were counted by light microscopy. The ART4 phenotype replicated across these three experimental repeats.
Randomization	No randomization was performed.
Blinding	For the functional analysis, slides were counted blind.

Reporting for specific materials, systems and methods

We require information from authors about some types of materials, experimental systems and methods used in many studies. Here, indicate whether each material, system or method listed is relevant to your study. If you are not sure if a list item applies to your research, read the appropriate section before selecting a response.

Materials & experimental systems

n/a	Involved in the study	
[ ]	[x]	Antibodies
[ ]	[x]	Eukaryotic cell lines
[x]	[ ]	Palaeontology and archaeology
[x]	[ ]	Animals and other organisms
[ ]	[x]	Human research participants
[x]	[ ]	Clinical data
[x]	[ ]	Dual use research of concern

Methods

n/a	Involved in the study	
[ ]	[x]	ChIP-seq
[ ]	[x]	Flow cytometry
[x]	[ ]	MRI-based neuroimaging

Antibodies

Antibodies used	Anti-CD49d PE-Violet 770 (Miltenyi Biotec), anti-CD36 Violet Blue (Miltenyi Biotec), anti-CD71 APC (Miltenyi Biotec), anti-basigin FITC (Invitrogen), anti-CD55 FITC (Miltenyi Biotec), anti-CD45-PB (BD bioscience), anti-CD16-FITC (BD bioscience), CD61-PE (eBioscence), CD41-FITC (eBioscience), anti-AQP1 (Invitrogen), anti-ART4 (AbNova), anti-N201 (mouse serum, doi:10.1074/mcp.M113.029272), anti-RON4 (mouse serum, doi:10.1128/EC.00040-06).
Validation	The target species for all commercial antibodies used was human. All commercial antibodies were validated by manufacturer for target species. In all cases human was the primary target species. Anti-RON4 and anti-N201 were validated in previous works (doi:10.1074/mcp.M113.029272 and doi:10.1128/EC.00040-06).

Eukaryotic cell lines

Policy information about cell lines

Cell line source(s)	Immortalized erythroid progenitor cell line (EJ cells) generated from peripheral blood mononuclear cells (https://doi.org/10.1002/ajh.25543). This cell line was generated in house within the Duraisingh lab.
Authentication	EJ cells were characterized as erythroid cells through light microscopy examination of cytospins post differentiation and surface expression of known erythroid markers as observed by flow cytometry.<br>CRISPR knock outs with the EJ background were verified by flow cytometry and TIDE software.
Mycoplasma contamination	Not tested.
Commonly misidentified lines (See ICLAC register)	N/A.

Human research participants

Policy information about studies involving human research participants

Population characteristics	Subjects are children aged 0-180 months, of both sexes, and belonging to Mossi ethnic group (self-reported ethnicity of both parents) from Burkina Faso.
Recruitment	The sample of severe malaria cases was recruited at the 158-bed paediatric ward of the Ouagadougou University Hospital. In line with WHO guidelines, severe malaria was defined by the presence of P. falciparum in the thick blood film associated with at least one of the following conditions: prostration (incapacity of the child to sit without help in the absence of coma), unrousable coma (score between 0 and 2 on the Glasgow modified coma scale), repeated generalised convulsions (more than two episodes in the preceding 24 h), severe anaemia (haemoglobin <5 g per 100 ml), hypoglycaemia (<40 mg per 100 ml), pulmonary oedema/respiratory distress, spontaneous bleeding and renal failure (plasma creatinine>3 mg per 100 ml). The sample of healthy control children was recruited during malaria cross-sectional surveys performed in the Ouagadougou area.
Ethics oversight	The study received approval from the ethical committees of the Ministry of Health of Burkina Faso and the University of Oxford.

Note that full information on the approval of the study protocol must also be provided in the manuscript.

ChIP-seq

Data deposition

☐ Confirm that both raw and final processed data have been deposited in a public database such as GEO.

☐ Confirm that you have deposited or provided access to graph files (e.g. BED files) for the called peaks.

Data access links<br>*May remain private before publication.*	*For "Initial submission" or "Revised version" documents, provide reviewer access links. For your "Final submission" document, provide a link to the deposited data.*
Files in database submission	*Provide a list of all files available in the database submission.*
Genome browser session<br>(e.g. UCSC)	*Provide a link to an anonymized genome browser session for "Initial submission" and "Revised version" documents only, to enable peer review. Write "no longer applicable" for "Final submission" documents.*

Methodology

Replicates	*Describe the experimental replicates, specifying number, type and replicate agreement.*
Sequencing depth	*Describe the sequencing depth for each experiment, providing the total number of reads, uniquely mapped reads, length of reads and whether they were paired- or single-end.*

Antibodies	*Describe the antibodies used for the ChIP-seq experiments; as applicable, provide supplier name, catalog number, clone name, and lot number.*
Peak calling parameters	*Specify the command line program and parameters used for read mapping and peak calling, including the ChIP, control and index files used.*
Data quality	*Describe the methods used to ensure data quality in full detail, including how many peaks are at FDR 5% and above 5-fold enrichment.*
Software	*Describe the software used to collect and analyze the ChIP-seq data. For custom code that has been deposited into a community repository, provide accession details.*

Flow Cytometry

Plots

Confirm that:

- [ ] The axis labels state the marker and fluorochrome used (e.g. CD4-FITC).
- [x] The axis scales are clearly visible. Include numbers along axes only for bottom left plot of group (a 'group' is an analysis of identical markers).
- [ ] All plots are contour plots with outliers or pseudocolor plots.
- [ ] A numerical value for number of cells or percentage (with statistics) is provided.

Methodology

Sample preparation	Day 8 ejRBCS were stained in 100µL of flow buffer (0.1% BSA in 1× PBS).
Instrument	For analysis of erythroid markers on EJ cells, MACSQuant Analyzer 10 flow cytometer (Miltenyi Biotec) was used. For evaluation of erythrocyte purty for proteomics, Gallios Flow Cytometer (Beckman Coulter) was used.
Software	For analysis of erythroid markers on EJ cells, FlowJo (version 10.4); Kaluza software v.2.0 (Beckman Coulter) for analysis of mature erythrocyte purity for proteomics.
Cell population abundance	For analysis of erythroid markers on EJ cells, 50,000 cells were acquired. Cell populations were separated by a live/dead stain (Propidium iodide;PI).<br>For erythrocyte analysis for proteomics, 500,000 events were acquired.
Gating strategy	Ej cells were gated FSA vs SCA; FSA vs FSH for singlets; FSA vs Propidium iodide (PI)- here two populations were present and cells negative for PI were selected; a histogram for the relevant antibody was plotted.

- [ ] Tick this box to confirm that a figure exemplifying the gating strategy is provided in the Supplementary Information.
